# Evaluating a complex health promotion program to reduce hepatitis C among Aboriginal and Torres Strait Islander peoples in New South Wales, Australia: the Deadly Liver Mob

**DOI:** 10.1186/s12954-023-00885-9

**Published:** 2023-10-20

**Authors:** Carla Treloar, Kim Beadman, Mitch Beadman, Kerri-Anne Smith, Jade Christian, Aunty Clair Jackson, Beverley Tyson, Clayton Anderson, Larissa Smyth, Melinda Walker, Jennifer Heslop, Gary Gahan, Victor Tawil, Felicity Sheaves, Louise Maher, Julie Page, Donna Tilley, Ann Ryan, Kim Grant, Basil Donovan, Annabelle Stevens, Trevor Slattery, Kate Pearce, Franklin John-Leader, Andrew Walden, Jo Lenton, Margaret Crowley, Elena Cama

**Affiliations:** 1 Centre for Social Research in Health, John Goodsell Building, UNSW Sydney, Kensington, NSW 2052 Australia; 2 Needle and Syringe Program, Mount Druitt Community Health Centre, Western Sydney Local Health District, Sydney, NSW 2770 Australia; 3 Needle and Syringe Program, Nepean Blue Mountains Local Health District, Penrith, NSW 2747 Australia; 4 Dubbo Sexual Health, Western NSW Local Health District, Dubbo, NSW 2830 Australia; 5 Byron Central Hospital, Mid North Coast and Northern NSW Local Health District, Byron Bay, NSW 2481 Australia; 6 HIV & Related Programs, Mid North Coast and Northern NSW Local Health District, Coffs Harbour, NSW 2450 Australia; 7 Kirketon Road Centre, South Eastern Sydney Local Health District, Sydney, NSW 1340 Australia; 8 Centre for Population Health, Ministry of Health, St Leonards, NSW 2065 Australia; 9 Western Sydney Sexual Health Centre, Western Sydney Local Health District, Parramatta, NSW 2150 Australia; 10 HIV & Related Programs (HARP) Unit, Western and Far West NSW Local Health District, Dubbo, NSW 2830 Australia; 11 Kirby Institute, UNSW Sydney, Sydney, NSW 2052 Australia; 12 Needle and Syringe Program, Western NSW Local Health District, Dubbo, NSW 2830 Australia; 13 Broken Hill Community Centre, Far West Local Health District, Broken Hill, NSW 2880 Australia

**Keywords:** Aboriginal and Torres Strait Islander people, Blood borne viruses, Health promotion, Hepatitis C, Sexually transmissible infections

## Abstract

The Deadly Liver Mob (DLM) is a peer-delivered incentivised health promotion program by and for Aboriginal and Torres Strait Islander Australians, and was introduced in response to the disproportionate number of Aboriginal and Torres Strait Islander Australians who are impacted by blood borne viruses (BBVs) and sexually transmitted infections (STIs). The goal of the program is to increase access to BBV and STI education, screening, treatment, and vaccination in recognition and response to the systemic barriers that Aboriginal and Torres Strait Islander peoples face in accessing health care. This commentary introduces a series of papers that report on various aspects of the evaluation of the Deadly Liver Mob (DLM) program. In this paper, we explain what DLM is and how we constructed an evaluation framework for this complex health promotion intervention.

This commentary is the first in a series of papers that report on various components of the evaluation of a complex health promotion program aiming to increase access to screening and treatment for blood borne viruses (BBVs) and sexually transmitted infections (STIs) among Aboriginal and Torres Strait Islander Australians. The Deadly Liver Mob (DLM) program ran in several sites in New South Wales, Australia from 2013 to 2020. In this paper, we explain what DLM is and how we constructed an evaluation framework using the RE-AIM framework [1]. It is important to provide this detail as we sought to work with concepts important to Aboriginal and Torres Strait Islander notions of holistic health encompassing physical, emotional, social, and spiritual aspects [2] and to explain the choices made in developing the evaluation framework that were informed by guidelines for complex health promotion interventions [3] but avoided replicating harmful colonising approaches [4].

In this paper we respectfully use Aboriginal and Torres Strait Islander to refer to the First Nations peoples of Australia. We use the term First Nations to refer to Indigenous peoples around the world.

## Why was DLM started?

It is well known that mainstream health services are, typically, not set up to serve the needs of First Nations people and can fail to attract and retain First Nations people in care. It is important that any analysis of barriers to health care is framed within a strengths-based approach which foregrounds social relations and collective practices and identities [5] and avoids deficit models of First Nations people, which tend to avoid examining structural and organisational barriers to engagement in mainstream health settings [6]. In an analysis of barriers and enablers to health care access among a range of Aboriginal and Torres Strait Islander communities in New South Wales (NSW), Australia, a number of themes have been identified, including: “coordination of healthcare services within jurisdictions, effective communication between healthcare services, trust in health services and positive experiences of cultural safety, prioritization of access for Aboriginal people, resourcing for healthcare services and addressing distance and transport barriers” [7] (p10).

Aboriginal and Torres Strait Islander Australians make up just 3.2% of the total Australian population [8], yet are disproportionately affected by BBVs and STIs. Notification rates for HCV are reported to be 5.9 times higher for Aboriginal and Torres Strait Islander Australians compared to non-Aboriginal and Torres Strait Islander Australians (167.3 and 28.5 per 100 000, respectively in 2020) [9]. Further, although HCV antibody prevalence has declined overall among respondents of the Australian Needle and Syringe Program Survey between 2018 to 2022, prevalence remains consistently higher among Aboriginal and Torres Strait Islander participants [10]. Notification rates for chlamydia, gonorrhoea, syphilis, hepatitis B virus (HBV), and HIV among Aboriginal and Torres Strait Islander people are diagnosed at 2.8, 4.2, 5.5, 1.8, and 1.6 times respectively the rates of non-Aboriginal and Torres Strait Islander people in Australia (rates per 100,000 in 2020) [9, 11]. In light of the disproportionate impact of BBVs and STIs, Aboriginal and Torres Strait Islander Australians are noted as priority populations in both New South Wales [12] and national strategies for BBVs and STIs [13].

DLM was designed to better support Aboriginal and Torres Strait Islander people to manage HCV, with recognition that offering effective testing and treatment is central to disease control [14]. The enablers identified above were key to re-designing services to be effective in engaging Aboriginal and Torres Strait Islander people in HCV testing and treatment.

## What is DLM?

Deadly Liver Mob (DLM) is a peer-driven, incentivized health promotion program that provides a culturally safe, sensitive, and appropriate way to increase access to testing and treatment of BBVs and STIs among Aboriginal and Torres Strait Islander Australians. It is important to note that the word “deadly” is used by Aboriginal and Torres Strait Islander Australians to indicate something that is very good or excellent [15]. The aims of the DLM program are to:

- Raise awareness about HCV, including transmission risk factors and treatment options;
- Increase access to BBV and STI testing and treatment by Aboriginal and Torres Strait Islander people; and
- Provide a point of entry to other health services for Aboriginal and Torres Strait Islander people.

A further goal of this project centered on establishing DLM as a sustainable program in routine operations of a health service. This is important as the cycle of piloting programs for First Nations people and then removing them can be dispiriting for communities [16].

DLM began as a trial program in one site in metropolitan Sydney in 2013. This expanded to a second site in the pilot phase. Results from this pilot have been published [17, 18]. On the strength of the pilot results, funding was sought and provided through a Partnership Project Grant by the National Health and Medical Research Council (NHMRC), with additional support from the NSW Ministry of Health, to implement DLM in an additional seven sites. The program was subsequently run in nine sites within seven Local Health Districts^1^ (LHDs) that covered inner city, regional and remote locations. The grant supported implementation and evaluation of each site to inform scale-up plans [19].

DLM uses a peer-driven intervention that asks Aboriginal and Torres Strait Islander community members to attend for education about viral hepatitis with an Aboriginal and/or Torres Strait Islander health worker, be screened for BBVs and STIs, return for their results, and receive any required treatment or vaccination. Clients are also asked to pass on their learnings to family and friends, and to encourage them to attend DLM. Each contact with the health service entitles a DLM client to an incentive payment in the form of a voucher. These contacts include: education, recruitment of others, attendance for screening, returning for results, and additional sexual health treatment or vaccination for hepatitis A or B (HBV) (if required) [17]. Given the high rates of attendance of Aboriginal and Torres Strait Islander people at Needle and Syringe Programs (NSPs) and the high rates of HCV among people who inject drugs, NSPs were designed to be the “front door” of the DLM project. At the time of program establishment, NSPs were not providing clinical services, such as screening. To facilitate the connection between education, testing, and treatment, a partnership was established with sexual health services that were co-located with NSPs on health campuses.

DLM is modelled on the Safe Injecting CWIZ (SIC) project that was conducted by the architects of DLM in 1998–2002 and targeted people under the age of 25 years who injected drugs [20]. The SIC project was an adaptation of HIV peer driven interventions for injecting drug users originating in the United States [21, 22]. These earlier projects provided the evidence to establish a focused, incentive-based education program that would reach deep into networks that were difficult for existing services to access. There is extensive evidence suggesting that incentives have benefits to health outcomes (e.g., see [23–27]).

The DLM program was designed to meet the needs of the community and to ensure that messages were culturally appropriate. Aboriginal and Torres Strait Islander cultures share stories through yarning, an Indigenous style of conversation and telling and sharing stories and information [28, 29]. The DLM model of yarning to clients about HCV and then asking them to pass on the messages to their peers was seen as a culturally effective approach. Critical to the success of the initial DLM program were the Aboriginal and Torres Strait Islander staff (sexual health workers and frontline NSP workers) who were central to co-design and implementation of the education materials and approach.

The education is guided by visual aids and conversational chunks of information such as: what is HCV?; how do I get it?; how do I avoid it?; what can I do if I have it? The education session is designed to be easily accessible for clients with low literacy. Yarning between the Aboriginal and Torres Strait Islander staff member and the DLM clint in HCV education sessions monitor the quality of peer messages and build on the client’s knowledge while dispelling any myths. Yarning takes possible negative experiences of education into account by taking a non-authoritative approach to knowledge building.

Following education, the DLM worker offered clients the opportunity for testing. The Aboriginal and/or Torres Strait Islander DLM worker accompanied the client to screening to introduce sexual health workers (many of whom are non-Aboriginal or Torres Strait Islander) who manage the clinical aspects of DLM including screening, delivery of results, and provision of treatment such as for HBV (vaccination or treatment), HCV, HIV, and STIs (if required) as per standard care. When initially introduced in two pilot sites in 2013 and 2015, direct-acting antiviral treatments for HCV had not yet been introduced, thus treatment available for HCV consisted of combination pegylated interferon-alpha with ribavirin. In March 2016, the Australian Government began subsidizing access to direct-acting antiviral treatments, which became the treatment available for HCV across existing and subsequent DLM sites. The DLM Aboriginal and Torres Strait Islanders workers were involved, if needed, in re-contacting DLM participants to ensure that test results could be delivered by clinical staff.

## Program logic as foundational to evaluation

**Fig. 1:**
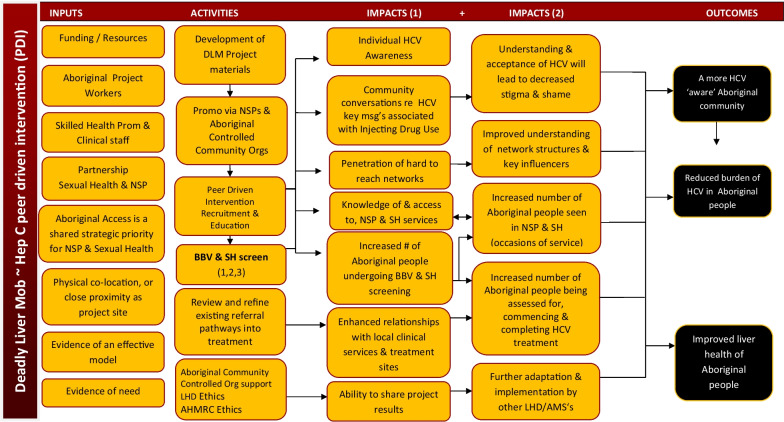
Program logic for Deadly Liver Mob

Robust and high-quality evaluation frameworks are built upon and examine the causal assumptions of the intervention [3, 30]. Figure 1 presents the causal assumptions [3, 30] of the DLM progam via a program logic display. The program logic illustrates the desired outcomes for the project and links these to the impacts, activities, and inputs required to establish and implement the DLM program, including: (1) Inputs: The model describes the range of resources that are required to establish and run a DLM program including funding (especially for designated Aboriginal and Torres Strait Islander workers and incentive payments), appropriate engagement and ownership of the program by Aboriginal and Torres Strait Islander health workers, and partnerships between services; (2) Activities: The model describes the activities required to successfully plan for implementation of the DLM program including development of educational activities to be provided to community members, promotion of the program to the local community, facilitating screening, preparing referral pathways between services and gaining approval from appropriate ethics committees; (3) Impacts: The impacts of the DLM program are considered in a range of areas including domains of knowledge, access to services, screening, decreasing stigma associated with HCV and service level impacts such as improved community relationships; and, (4) Outcomes: Following the aims of the program, the projected outcomes of DLM programs relate to increased awareness of HCV among Aboriginal and Torres Strait Islander communities as well as increased uptake of BBV and STI testing and treatment and overall improvement in liver health of the target community. Building the DLM evaluation framework involved mapping elements of the program logic to relevant aspects of the RE-AIM framework, as outlined below.

## Designing the evaluation of a complex health promotion program

In designing the DLM evaluation framework, we used insights from the RE-AIM model [1] and from the model developed by the UK Medical Research Council for process evaluations of complex interventions [3]. These models are appropriate to DLM as we were seeking to understand real-world application of a new service model using a low-threshold evaluation framework. Low threshold evaluation is important as we considered it unethical for the evaluation requirements to produce any further barriers to health care access for Aboriginal and Torres Strait Islander people. For example, this evaluation operated under a consent waiver. That is, consent from individuals was not sought for use of existing routine data sources, an approach approved through institutional ethics. Further, as Moore and colleagues note [3], process evaluation beyond pilot stages is important to examine issues in implementation in more diverse settings and contexts. DLM grew from implementation in one and then two sites in a pilot program to seven additional sites (with additional outreach activities). Further, focusing the evaluation on issues of adaptation and context in relation to fidelity was important to inform roll-out in future sites.

### Is this a complex intervention?

Moore et al. [3] define complex interventions as comprising multiple interacting components that typically undergo some tailoring for different contexts. DLM comprised education, testing, and potentially treatment components delivered by Aboriginal and Torres Strait Islander and non-Aboriginal and Torres Strait Islander workers in co-located but distinct health services (one site had an integrated model of care). The core elements of DLM were defined as: led by an Aboriginal and/or Torres Strait Islander worker, co-located services, incentives, yarning-based education, and warm and facilitated referral from an Aboriginal and Torres Strait Islander worker to a (typically) non-Aboriginal and Torres Strait Islander worker for clinical services and care. The ways in which DLM was implemented in each site was adapted to suit context and will be explored under the Implementation dimension of RE-AIM.

### Staffing the evaluation

The research funds from the NHMRC enabled the establishment of an independent evaluation team based at the Centre for Social Research in Health at UNSW Sydney. As Moore and colleagues note [3], there is the need to balance good and close relationships with staff at DLM sites (enabling observation of elements of adaption and fidelity) with independence to allow critical analysis and evaluation of program operations.

DLM operated as a network with governance and processes established to guide both implementation and evaluation. A particular issue in governance processes was the involvement of Aboriginal and Torres Strait Islander people to meet the expectations of community control in delivery and in evaluation of the DLM program. The governance policies emphasised the need for Aboriginal and Torres Strait Islander people to be involved in all aspects of the program in implementation, evaluation, and in public presentation of the DLM program.

The nine sites involved in the DLM program committed to regular teleconference meetings for management and for catch-up with sites and an annual face-to-face meeting, which included opportunities for the Aboriginal and Torres Strait Islander workers to meet. These processes enabled the staff at each site to share information about the ways in which they had solved implementation issues at their site and for the evaluation team to monitor and record these issues. These meetings provided the opportunity for the evaluation team to identify instances where data were recorded in different ways in some sites and to harmonise these in a timely way.

The plan for the evaluation design included qualitative data (interviews with DLM clients and Aboriginal and Torres Strait Islander and non- Aboriginal and Torres Strait Islander staff), quantitative data (routinely collected data from NSP services) and data linkage (with data extractions from the specific sexual health services involved via a sentinel surveillance program [31]). In addition, the evaluation team managed a register of issues and solutions (related to implementation and adaptation) which were used to inform interview schedules for qualitative data collection and in the development of an online implementation toolkit (see www.deadlylivermob.org). The next section illustrates how the evaluation framework was designed using the RE-AIM framework to examine the causal assumptions of the DLM program (as illustrated in the program logic).

## The RE-AIM framework for evaluating DLM

The five dimensions of the RE-AIM framework were designed to guide more comprehensive evaluations of public health programs designed for wide-scale and real-world implementation, such as in busy services operating without the support of a research infrastructure. Part of the vision of RE-AIM is to move beyond individually focused outcome measures, to those which reach into organisational levels. That is, RE-AIM guides evaluations to consider factors beyond only efficacy, and to incorporate measures and evaluation designs that broaden the range of factors beyond clinical measures only. In this case, the acceptability of the DLM to clients and staff was also a key aspect of evaluation. The sections below map out the key evaluation aims of each element of RE-AIM and then how the DLM evaluation framework was designed to examine these.

### Reach

In this element of the framework, evaluations should consider the individual characteristics of DLM participants in comparison with the broader target population. The target population for DLM included Aboriginal and Torres Strait Islander people living, working, or visiting the area around the DLM program and those who had ever or currently injected drugs, or were classified as “at risk” of injecting drug use and/or BBVs and/or STIs (such as history of incarceration, unsafe tattoo, or living with a person who inject drugs and/or who has HCV). DLM sites were established in areas where significant numbers of Aboriginal and Torres Strait Islander people were known to live. Many DLM sites established outreach work where they would conduct the DLM program in other services which potential DLM participants were already accessing (such as housing, probation and parole, and in community locations). The provision of incentives included the rationale that this small amount of money could support the attendance of people who might not otherwise have the means to attend, including covering transport costs.

#### Evaluation design for reach

In a commitment to low threshold access to DLM, minimal data about DLM participants was collected directly. We were able to use data routinely collected by the service on participants’ engagement in the various stages of the program, which also included peer referral or how many DLM participants recruited others to the program. If DLM participants accessed clinical services, we were able to use data linkage to explore additional demographic and clinical variables. Qualitative research (for which individual consent was obtained) explored issues around reach, including barriers to engagement and participation.

### Efficacy

This is the second individual level dimension of the RE-AIM framework and is focused on determining whether the program achieved its aims. The authors of the RE-AIM framework also point to the need to consider not just biological but also behavioural (across all actors in the program) and participant-centred indicators of program impact (such as broader indicators of well-being and satisfaction with the program). Evaluation of efficacy should also focus on positive and negative outcomes including unexpected pathways and consequences [3].

#### Evaluation design for efficacy

The outcomes within the DLM program logic model include increased awareness of HCV and decreased burden of HCV among Aboriginal and Torres Strait Islander communities via linkage to testing and treatment. It was considered that assessment of knowledge of HCV via survey of DLM participants was not appropriate. For example, a survey of DLM participants prior to engagement in DLM (and with associated informed consent procedures) would have presented an unacceptable and unethical barrier to service access. Further a survey of knowledge would risk being considered by clients as researchers working within a deficits-based approach (that is, looking for gaps in knowledge that needed to be remedied) rather than framing the project as considering how health services could more effectively communicate health information to Aboriginal and Torres Strait Islander clients. In qualitative interviews, we explored what DLM clients valued about the program including what knowledge was gained and whether being a part of DLM affected their engagement with HCV and other health services. These interviews were conducted by Aboriginal researchers from the evaluation team and not involved in DLM operations or clinical care. Similarly, in qualitative interviews with DLM staff, we explored their perceptions of the impact of DLM on clients’ health care access, as well as their perspectives of offering the DLM program (and associated services) including implementation and adaptation issues. Using data linkage, we planned to explore clinical outcomes relevant to DLM efficacy including proportion of new diagnoses, treatments provided in the local region. In addition, we planned to establish whether a DLM participant had either accessed sexual health services prior to DLM, and whether they returned for additional care following engagement in DLM.

### Adoption

As an organisational-level RE-AIM dimension, adoption refers to the proportion and representativeness of settings that have adopted the program. Which settings have adopted the program can be observed.

#### Evaluation design for adoption

At full operation, DLM was operating in nine sites across the state of NSW (with additional outreach activities) which were situated in seven of 15 LHDs in the state. To further promulgate the DLM method and provide resources for additional sites, we have developed an online toolkit for implementation (see www.deadlylivermob.org).

### Implementation

The implementation dimension comprises both individual and organisational levels and refers to the extent to which the program was delivered as intended to explore which factors of an intervention are practical and feasible to deliver in everyday practice.

#### Evaluation design for implementation

The delivery of DLM was a balance between fidelity to program as designed and the need for adaption in line with resources, opportunities, and limitations of each site [3]. To understand the ways in which the DLM program was implemented in the first 6–12 months in each site, we worked closely with each site to document ways in which the core principles of the DLM intervention were delivered and adapted when necessary. We collected information during governance, site-catch up, and annual face-to-face meetings, and logged these in an issues register. For example, the provision of incentives was originally structured as AUD $20 for education and AUD $10 for each subsequent engagement. However, some regionally based services with less public transport infrastructure and greater distance required for travel, amended the incentives to provide AUD $20 for return visits and reduced the incentive provided for initial education. In this way, the principle of DLM was maintained as intended (offering of incentives and at a capped total amount) and that adaption was necessary to meet local needs of transport costs to serve the greater mission of facilitating access to services for people from marginalised groups. In another example, sites differed in relation to their capacity to run DLM programs two days or one day a week. This created a difference in relation to dose (and reach) but did not challenge fidelity as the agreed core aspects of the program were implemented. These ways in which the different DLM programs managed implementation (at establishment and over time) were synthesised and presented in the online implementation toolkit, emphasising the need for and the ability of new DLM sites to use the framework of DLM and maintain fidelity to its goals, within the opportunities and constraints of each new location.

### Maintenance

The RE-AIM framework draws attention to long term maintenance of programs; that is, for more than two years with analysis using both individual and organisational levels of focus. DLM was supported by a larger evaluation project initially planned to run for three years from 2017 to 2020 (extended into 2021 because of COVID-19 disruptions to service provision) with the expectation that a significant amount of time was required for a program of this type to become institutionalised as routine programming and supported by management.

#### Evaluation design for maintenance

While DLM was supported for a three-year evaluation, there were factors that significantly impacted operations. Data collected in team meetings and via the issues register were used to map interruptions to service (such as not having an Aboriginal and Torres Strait Islander worker available to conduct DLM for a period). We used these data to populate ‘trouble shooting’ sections of the implementation toolkit to guide future sites in strategising solutions to maintain effective connection with Aboriginal and Torres Strait Islander communities. The final period of DLM evaluation overlapped with the start of the COVID-19 pandemic. Given the vulnerabilities of Aboriginal and Torres Strait Islander communities to COVID-19 [32], many non-essential health services were suspended in this period, including DLM operations. While the COVID-19 epidemic stabilised in NSW during the latter part of 2020, numerous outbreaks occurred across 2021 (including a lockdown exceeding three months) making the stabilisation of DLM programs difficult to assess. We also note that the principles of DLM were adapted for use in non-Aboriginal and Torres Strait Islander communities who could benefit from connection to HCV education, testing, and treatment via needle and syringe programs [33].

## Conclusion

Evaluation of real-world programs which are designed to improve the health of marginalised groups requires a careful design with a sensitive and flexible research apparatus. The main goal of the evaluation design was not to interfere with the program’s efforts to engage Aboriginal and Torres Strait Islander people in health care. This meant, for example, negotiating a waiver of consent to use routinely collected data that can be messy and vary across settings, using qualitative data to explore the detail of program delivery and acceptability, and using linked data to explore clinical outcomes of the program (with necessary delays and quality issues to navigate). This evaluation design also necessitated close engagement between researchers and those involved in program delivery as we sought to understand the various ways in which sites implemented DLM. However, this engagement brings a richness to the evaluation process and its outputs, particularly in the design of a toolkit to enable others to implement this program as efforts to improve the health of Aboriginal and Torres Strait Islander people must continue, and critically engage with the key barriers that have produced such stark inequities in health over centuries of colonisation.

## Data Availability

The datasets generated and/or analysed during the current study are not publicly available due to the possibility of individual privacy being compromised.

## Abbreviations

BBV: Blood-borne virus
DLM: Deadly liver mob
HBV: Hepatitis B
HCV: Hepatitis C
LHD: Local Health District
NHMRC: National Health & Medical Research Council
NSP: Needle and syringe program
NSW: New South Wales
SIC: Safe injecting CWIZ
STI: Sexually transmissible infection

## Footnotes

1 Local Health Districts (LHDs) manage any public hospitals and health services within defined geographical areas in NSW. There are currently 15 Local Health Districts across the state; eight covering the Sydney metropolitan area and seven covering rural and regional NSW (ref https://www.health.nsw.gov.au/lhd/Pages/default.aspx).
